# Primates Living Outside Protected Habitats Are More Stressed: The Case of Black Howler Monkeys in the Yucatán Peninsula

**DOI:** 10.1371/journal.pone.0112329

**Published:** 2014-11-06

**Authors:** Ariadna Rangel-Negrín, Alejandro Coyohua-Fuentes, Roberto Chavira, Domingo Canales-Espinosa, Pedro Américo D. Dias

**Affiliations:** 1 Instituto de Neuroetología, Universidad Veracruzana, Xalapa, Veracruz, Mexico; 2 Instituto de Ciencias Médicas y Nutrición Salvador Zubirán, México D.F., Mexico; German Primate Centre, Germany

## Abstract

The non-invasive monitoring of glucocorticoid hormones allows for the assessment of the physiological effects of anthropogenic disturbances on wildlife. Variation in glucocorticoid levels of the same species between protected and unprotect areas seldom has been measured, and the available evidence suggests that this relationship may depend on species-specific habitat requirements and biology. In the present study we focused on black howler monkeys (*Alouatta pigra*), a canopy-dwelling primate species, as a case study to evaluate the physiological consequences of living in unprotected areas, and relate them with intragroup competition and competition with extragroup individuals. From February 2006 to September 2007 we collected 371 fecal samples from 21 adults belonging to five groups (two from protected and three from unprotected areas) in Campeche, Mexico. We recorded agonistic interactions within groups and encounters with other groups (1,200 h of behavioral observations), and determined fecal glucocorticoid metabolite (FGM) concentrations with radioimmunoassays. We used linear mixed models and Akaike's information criterion to choose the best model explaining variation in FGM concentrations between protected and unprotected areas calculated from five categorical variables: habitat type (protected vs. unprotected), participation in agonistic interactions, intergroup encounters, sex and female reproductive state, and season. The best model included habitat type, the interaction between habitat type and agonism, and the interaction between habitat type and season. FGM concentrations were higher in unprotected habitats, particularly when individuals were involved in agonistic interactions; seasonal variation in FGM concentrations was only detected in protected habitats. High FGM concentrations in black howler monkeys living in unprotected habitats are associated with increased within-group food competition and probably associated with exposure to anthropogenic stressors and overall food scarcity. Because persistent high GC levels can be detrimental to health and fitness, populations living in disturbed unprotected areas may not be viable in the long-term.

## Introduction

The effectiveness of protected areas to preserve biodiversity has been questioned [1], despite the prominence of protected areas as a cornerstone of current conservation efforts. Throughout the world, land clearing, logging, fires, hunting and grazing are less pronounced inside protected areas than in their surroundings [2], indicating that protected areas represent refuges for many threatened species and natural ecosystem processes [3]. The factors with the most obvious negative consequences on wildlife persistence in unprotected areas are loss of natural habitat and hunting. However, in these areas wildlife faces numerous deterministic and stochastic threats that affect their biology and behavior, and may lead to population decline and extinction [4]. For instance, habitat disturbance, such as habitat loss, results in shorter breeding seasons in blue tits (*Parus caeruleus*) [5], altered home ranges in greater gliders (*Petauroides volans*) [6], and increased food competition in Tana mangabeys (*Cercocebus galeritus*) [7]. In this context, understanding the physiological mechanisms underlying the responses of wildlife to anthropogenic disturbances may be instrumental to predict its persistence in unprotected areas.

The non-invasive sampling of glucocorticoid (GC) hormones allows researchers to assess the physiological effects of anthropogenic disturbances on wildlife [8]. The modulation of GCs is part of the adaptive physiological stress response, and these hormones are involved in diverse actions [9]. GCs may alter an organism's response to an ongoing stressor and may prepare an organism's response to a subsequent stressor. Independently from the nature of the stressful stimuli, during the stress response, GCs increase circulating glucose through a number of mechanisms, contributing to the depletion of present and, when the action of a stressor is prolonged, future energy stores [9]. When GC levels remain elevated for days or weeks they may become detrimental to health and fitness [10]. Anthropogenic disturbances, such as pollution, exposure to humans, and human-induced habitat transformation, elicit changes in GC levels in a variety of species [11]. For instance, GCs increase in: Magellanic penguins (*Spheniscus magellanicus*) exposed to oil following a petroleum spill [12]; wolves (*Canis lupus*) and elk (*Cervus elaphus*) that live in areas with high vehicle traffic [13]; and in northern spotted owls (*Strix occidentalis caurina*) that live in areas with timber harvesting [14]. However, variation in GC levels of the same species between protected and unprotected areas seldom has been measured, and the available evidence suggests that such variation may depend on species-specific habitat requirements and biology. For instance, whereas in hyenas (*Crocuta crocuta*), lions (*Panthera leo*) and maned wolves (*Chrysocyon brachyurus*) GC levels of individuals increase with increasing anthropogenic pressures in unprotected areas [15]–[17], there is no statistically significant difference in GC levels between elephants (*Loxodonta africana*) living in protected areas and in community conservation areas where human pastoral settlements and livestock grazing occur [18].

In the present study, we focused on the Yucatan black howler monkey (*Alouatta pigra*; hereafter, black howler monkeys) to evaluate the physiological consequences of living in unprotected areas, and relate them to changes in behavior. Black howler monkeys are tree-dwelling primates with a geographic distribution restricted to the Yucatan Peninsula in Mexico and Belize, and some parts of northern and central Guatemala [19]. In 2003, the International Union for Conservation of Nature conservation status of this species was revised from Insufficiently known to Endangered due to habitat loss and better available information [20]. There is evidence that habitat loss, hunting and continued presence of humans have a negative effect on the biology and behavior of black howler monkeys. In disturbed habitats, black howler monkeys: 1) live at densities up to five times higher than in extensive forests [21]; 2) search for food sources outside their habitats by walking on the ground, where they may be predated by domestic dogs and killed during road crossings [22], [23]; 3) are hunted for food and pet trading [24], [25]; and 4) show increased GC levels [26]. If black howler monkeys living in unprotected habitats show increased GC levels (associated with frequent exposure to anthropogenic stressors) and competition for resources (associated with more individuals living in smaller forests) their long-term presence in these habitats could be compromised due to reduced reproduction, immune function, and survival [27]. Therefore, the assessment of GC levels and competition levels in black howler monkeys may be very informative for the development of conservation policies involving populations of this endangered species.

We compared fecal GC metabolite (FGM) levels between black howler monkeys living in small unprotected forest fragments and black howler monkeys living in nearby extensive protected areas. We hypothesized that individuals living in unprotected forests would have higher FGM levels due to increased physiological stress associated with anthropogenic activities compared to individuals in protected areas. Because in forest fragments population densities are higher and the availability of food resources for howler monkeys is lower [28], we further hypothesized that higher FGM concentrations of black howler monkeys living in unprotected habitats would be associated with increased within- and between-group competition. Besides habitat type, within- and between-group competition, we examined whether the effects of sex, female reproductive state and environmental seasonality on FGM variation (which have been demonstrated to consistently affect the physiological stress response) [29], [30] varied according to habitat type.

## Methods

Our research complied with the Mexican law and was approved by the corresponding authorities (SEMARNAT SGPA/DGVS/01273/06 & 04949/07); and complied with the Guidelines for the Treatment of Animals in Behavioral Research and Teaching from the Animal Behavior Society.

### Study Area

We focused on populations of black howler monkeys living in the state of Campeche in Mexico. In Campeche the climate is hot and humid, and mean annual rainfall is 1,300 mm, with a drier season from November to May (mean monthly rainfall ± SD  = 43.7±25.8 mm), and a wetter period between June and October (218.9±14.1 mm). Mean annual temperature is 26°C [31].

**Figure 1:**
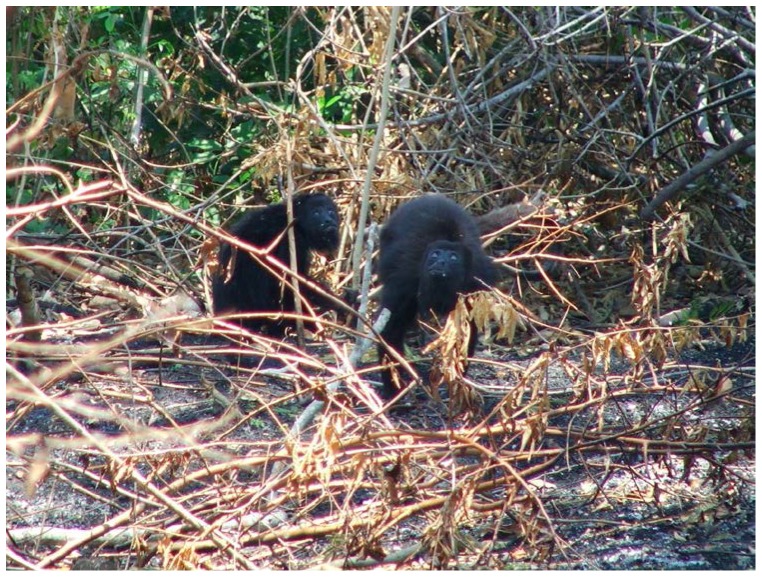
Black howler monkeys foraging on the ground in a recently burned forest fragment.

Campeche has a total area of 57,924 km^2^, from which approximately 40% is protected. There are black howler monkeys in two of the three larger protected areas in Campeche, the Calakmul Biosphere Reserve (18°19'00.28" N, 89°51'28.92" W) and the Laguna de Términos Reserve (18°51'15.38" N, 91°18'41.70" W). Together, these encompass an area of 14,282 km^2^. Although human activities occur in these reserves (e.g., apiculture, fishery), habitat availability is high for black howler monkeys and they face low anthropogenic stressors (e.g., hunting) [32]. We studied one group of black howler monkeys in each of these reserves (hereafter, protected habitat; Table 1). In contrast, the remaining non-urban territory of Campeche consists of highly humanized landscapes, where original habitats have been converted into forest-agricultural mosaics. Black howler monkeys living in these landscapes occupy forest fragments of variable size where they face multiple anthropogenic stressors on a daily basis that are generally absent in protected habitats, such as livestock grazing, predation threat by domestic animals or forest fires associated with slash-and-burn agriculture [23] (Figure 1). We studied three groups of black howler monkeys (Rancho El Álamo: 18°48'45.44" N, 90°58'54.61" W; ejido Chicbul: 18°46'51.66" N, 90°56'13.45" W; ejido General Ignacio Gutiérrez: 18°54'6.58" N, 90°53'37.90" W) living under these circumstances in three different forest fragments with areas <1 km^2^ (hereafter, unprotected habitat; Table 1). Mean (±SD) group size (7.1±0.5 individuals) and composition (adult males: 1.5±0.5; adult females: 2.3±0.7; immatures: 3.3±1.1) were very similar across groups (Table 1). All groups were habituated to human observers before the beginning of systematic behavioral and fecal sampling. Habituation consisted on the presence of three to four researchers near the group for a total of five days each week for two weeks (*ca*. 30 h). During habituation, researchers performed the same activities that would be performed during the study and observed whether animals reacted to them. We did not observe flights, avoidance, curiosity or displays directed towards researchers during habituation.

**Table 1:** Characteristics of the groups and habitats of black howler monkeys that were studied in unprotected and protected areas in Campeche, Mexico.

	Unprotected habitat			Protected habitat	
	Álamo	Chicbul	I. Gutiérrez	Calakmul	Términos
Area (km^2^)	0.96	0.05	0.02	7231.9	7061.5
Distance to nearest village (m)	1,640	771	1,092	27,000	18,100
Population density (ind/km^2^)	52	120	63	15.2	19.2
Group size *	6.4	7.7	6.7	7.3	7.3
Number of adult males *	1.5	1	2	2	1
Number of adult females *	2	2	3	1.5	3
Sampling months (dry/wet season samplings)	January/August	January/August	March/July	February/August	February/July

*Calculated as mean number of individuals during the two sampling periods (i.e., rainy and dry season).

### Behavioral data collection

To study within-group feeding competition, from February 2006 to September 2007 we recorded all occurrences of agonistic interactions (displacements, threats, chases and fights) in feeding context, defined as any interaction exchanged among adult individuals (*N* = 21; 12 females and nine males) when at least one of the individuals fed in a 10 min period before or after the interaction. When there was a latency of ≤5 seconds between two interactions of the same type, only one interaction was recorded. The sampling of social interactions was performed by a single observer (A. R.-N.) during complete day follows (i.e., 6:00–7:00 to 17:00–18:00, depending on the time of year). High spatial cohesion of howler monkey groups [33] allows for the observation of all individuals at the same time at any given point in time, and we therefore assume that we were able to sample adequately social interactions that occurred in feeding context. We also recorded encounters (both visual and vocal) with extragroup individuals to study intergroup competition. In each group, behavioral sampling was performed for five days a week (Monday to Friday) during four weeks in each season. A total of 30 sampling hours were collected each week, resulting in 240 sampling hours per group.

Following Dias et al. [34] and Van Belle et al. [35], we classified females as pregnant (defined as the 6 months preceding parturition), lactating (defined by either observations of lactation, or, as starting from the day of parturition until 15 months), or in other reproductive state (neither pregnant nor lactating). We based this classification on observations of births and lactation during the study and during periodical visits to the study groups up to 7 months after the end of the study.

### Fecal sample collection and hormone analyses

Fecal samples were collected opportunistically during behavioral samplings whenever they could be matched with individuals. Fresh samples uncontaminated by urine were collected from the forest floor and deposited in polyethylene bags labeled with the identity of each individual. Based on the estimated retention time of the digesta in the gut of *Alouatta pigra* of *ca*. 35 h [36], in order to match the occurrence of social interactions with the FGM levels of *Alouatta pigra* in each sampling week, we collected fecal samples from Tuesdays to Sundays. We analyzed 371 fecal samples (129 from individuals in protected habitats and 242 from individuals in unprotected habitats), with an average (± SD) of 17.7±6.5 samples per individual, 3.2±1.2 samples per week per individual and 9.8±2.9 samples per individual per season. Fecal samples were kept in a cooler with frozen gel packs while in the field and stored at the end of the day in a freezer at −20°C at the field station until extraction was performed following the methods described by Rangel-Negrín *et al*. [37].

FGM assays were conducted at the Instituto de Ciencias Médicas y Nutrición Salvador Zubirán, in Mexico City. We used a radioimmunoassay, a commercial ^125^I cortisol kit (SIEMENS Coat-a-count Cortisol), and a gamma counter (Cobra 5005, Packard Inc., MI, USA) to measure FGM levels in all samples.

As a biological validation of our assays, we determined the short-term effect of capture (an acute stressor) and anesthesia (ketamine) on the FGM excretion profile of three black howler monkeys (one male and two females) following the samples collection and conservation procedures described above. We collected all fecal samples (*n* = 42; 14±1 SD samples per individual) from 72 h before to 96 h after capture, and compared pre-capture levels with peak concentrations (i.e., the highest post-stressor values that were ≥2*SD above the mean concentration before capture) with a Wilcoxon signed-rank test. FGM levels peaked at a mean (± SD) of 35.3±9.9 h after capture. Peak FGM levels were significantly higher than pre-capture levels for the three individuals (female 1: *Z*_13_ = 2.27, *p*<0.05; female 2: *Z*_14_ = 3.29, *p*<0.001; male: *Z*_15_ = 3.41, *p*<0.001), indicating that our FGM assays reliably measured adrenal responses of black howler monkeys to stressors. Our capture and handling procedures [38] were approved by Mexican authorities (SEMARNAT, SGPA/DGVS/01273/06 & 04949/07).

Howler monkeys' pooled fecal extracts, when added to the standard curve points, exhibited an accuracy of *R* ^2^ = 0.99 (*n* = 5, *p* = 0.004), and serial dilutions of a fecal pool from howler monkeys yielded results that ran parallel to the FGM standards (*R* ^2^ = 0.97, *n* = 5, *p*<0.001). Samples were run in the order in which they were collected in a total of 58 assays, with a new set of quality controls beginning with assay 27. Cortisol intra-assay variation averaged 6.8% (fecal extract pool, *n* = 6). Inter-assay variation, estimated for the 58 assays from fecal pools with varying levels of cortisol, averaged 19.3% (low), 15.1% (medium), and 7.2% (high). All samples were run in duplicate, and mean FGM values are reported as ng/g (dry feces).

### Statistical analyses

We calculated weekly individual participation in agonistic interactions, participation in intergroup encounters (i.e., participated vs. did not participate), and mean FGM levels. Four individuals emigrated from our study groups from the first to the second sampling period (one in El Álamo and three in Calakmul). Therefore, our analyses were performed on 152 individual weeks.

To assess consistency within habitat types in individual variation in FGM levels, we compared the first with the last fecal sample collected for each individual in each season with a nonparametric Wilcoxon signed rank test.

We used linear mixed models (LMM) to assess the effects of five categorical variables on FGM levels: habitat type, participation in agonistic interactions (i.e., within-group competition), encounters with other groups (i.e., between-group competition), sex/female reproductive state and season. Because we were specifically interested in assessing the effects of habitat type on FGM levels, variables were included in the models as their interaction with habitat type (e.g., sex/female reproductive state x habitat type, season x habitat type). As the same individuals were repeatedly sampled, we included individual identity as a random factor in the model with first-order autocorrelation as a covariance structure. We used Akaike's information criterion (AIC) to choose the best model (i.e., lowest AIC: [39]). Post-hoc exploratory analyses were conducted on the effects of the interaction between habitat type and categorical within-group competition, and the interaction between habitat type and seasonality on FGM levels. For these analyses we used LMMs, in which pairwise combinations of habitat type x within-group competition and habitat type x seasonality were included as predictive fixed factors and individual identity was included as a random factor. FGM levels were normalized via logarithmic (ln) transformation. We checked that the assumptions of normally distributed and homogeneous residuals were fulfilled. All LMM analyses were performed in SPSS 22.0 (SPSS, Chicago, Illinois, U.S.A.).

## Results

There was no significant difference within seasons between the first and last FGM concentration measured for each individual in protected (rainy, *Z*_6_ = 0.524, *p* = 0.601; dry, *Z*_9_ = 1.01, *p* = 0.314) and unprotected habitats (rainy, *Z*_9_ = 1.125, *p* = 0.260; dry, *Z*_9_ = 0.415, *p* = 0.678).

Descriptive data on rates of within-group agonistic interactions and encounters with extragroup individuals are presented in Table 2.

**Table 2:** Mean (± SE) rates of within-group agonistic interactions and encounters with extragroup individuals for groups of black howler monkeys in unprotected and protected areas in Campeche, Mexico.

Habitat type		Weekly rates of agonistic interactions	Weekly rates of encounters with extragroup individuals
Unprotected	Álamo	0.021 (±0.003)	0.033 (±0.039)
	Chicbul	0.045 (±0.009)	0.004 (±0.031)
	I. Gutiérrez	0.052 (±0.013)	0.007 (±0.025)
Protected	Calakmul	0.034 (±0.006)	0 (±0.0)
	Términos	0.057 (±0.009)	0 (±0.0)

### Factors contributing to increased FGM levels

Our final model (*F*_5,143_ = 4.683, *p* = 0.001) included habitat type (*F*_1,18.220_ = 7.504, *p* = 0.013), the interaction between habitat type and agonistic interactions (*F*_2,134.866_ = 5.104, *p* = 0.007) and the interaction between habitat type and season (*F*_2,134.384_ = 3.937, *p* = 0.022; Table 3). The interaction between sex/female reproductive state and habitat type, as well as encounters with extragroup individuals were not selected.

**Table 3:** Parameter estimates for the best model explaining variation in FGM concentrations between groups of black howler monkeys living in protected and unprotected habitats in Campeche, Mexico.

Response variable	Estimate	SE	d.f.	*t* ratio	*P*	Confidence intervals	
						*Upper limit*	*Lower limit*
Intercept	2.42	0.05	56.95	51.177	<0.001	2.32	2.51
Habitat type	0.00	0.06	64.81	0.006	0.995	−0.13	0.13
Habitat type [non-protected]*agonism	0.15	0.05	140.36	3.112	0.002	0.06	0.25
Habitat type [protected]*agonism	0.05	0.07	129.79	0.723	0.471	−0.08	0.18
Habitat type [non-protected]*season	−0.04	0.05	127.39	−0.718	0.474	−0.13	0.06
Habitat type [protected]*season	−0.17	0.06	142.19	−2.713	0.007	−0.29	−0.05

**Figure 2:**
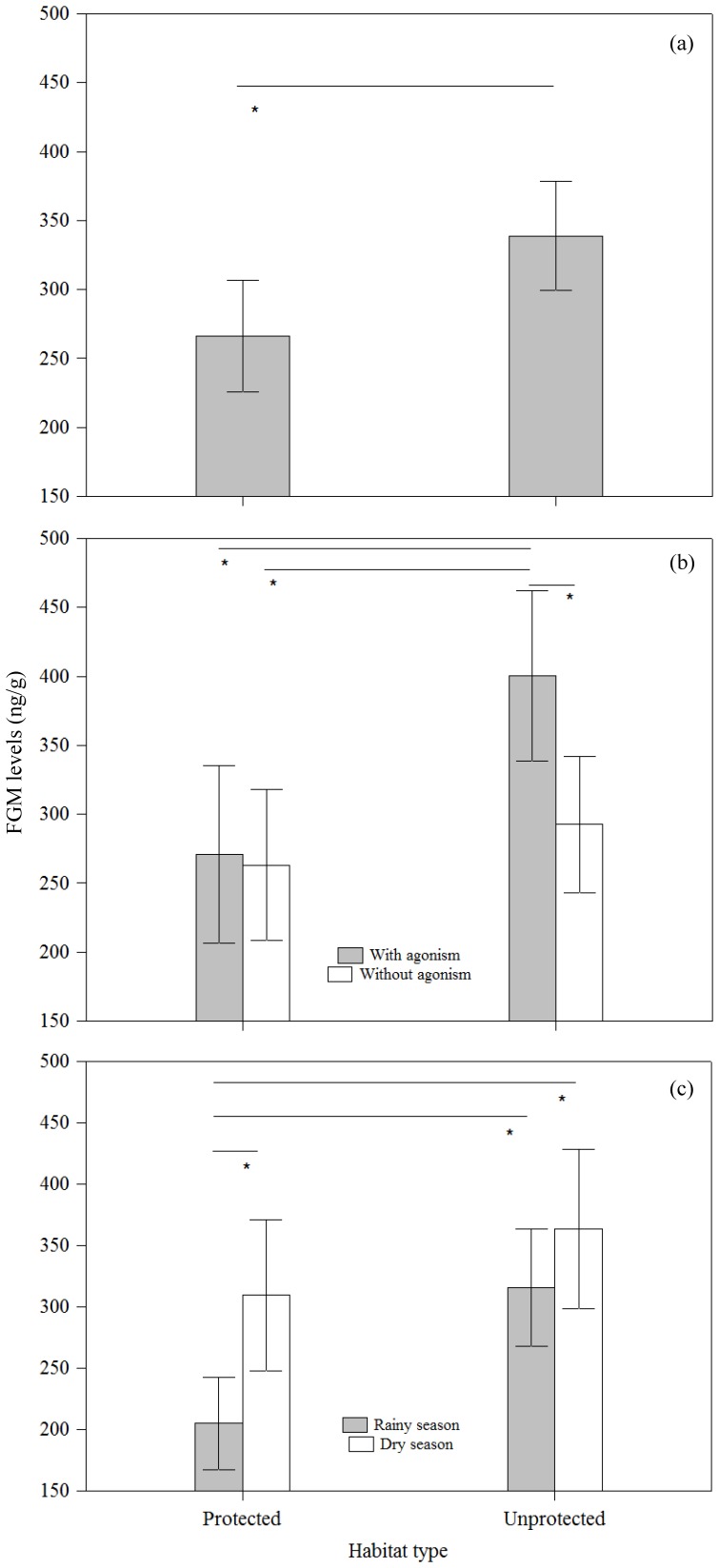
Variation in mean (±SE) FGM (fecal glucocorticoid metabolite) levels as a function of: a) habitat type; b) participation in agonistic interactions per habitat type; c) season per habitat type. Significant differences are denoted by an asterisk.

Overall mean FGM levels (± SE) of individuals living in unprotected habitats were approximately 20% higher (338.9±19.9 ng/g) than those of individuals living in protected habitats (266.2±20.3 ng/g; Figure 2a).

Agonistic interactions among group members occurred at similar proportions during sampling weeks in both habitat types (protected: 57.1% of individual/weeks; unprotected: 62%). However, when individuals living in unprotected habitats participated in agonistic interactions they had significantly (all post-hoc pairwise LMM *p*<0.01) higher mean (± SE) FGM levels (400.7±30.5 ng/g) than when they were not involved in such interactions (292.7±24.6 ng/g), or than individuals living in protected habitats (involved in agonistic behavior: 271.1±30.9 ng/g; non-involved in agonistic behavior: 263.3±26.9 ng/g; Figure 2b).

FGM levels (± SE) of individuals living in protected habitats (204.9±18.3 ng/g) were significantly lower (all post-hoc pairwise LMM *p*<0.05) during the rainy season than during the dry season (309.5±30.2 ng/g) and were lower than in both seasons in unprotected habitats (rainy: 315.8±23.9 ng/g; dry: 363.8±32.2 ng/g; Figure 2c).

## Discussion

Variation in FGM levels of black howler monkeys was significantly explained by the variables that were examined. In general, variation in FGM concentrations was highly consistent within each habitat type, and as predicted, FGM levels were higher in individuals living in unprotected habitats, particularly when they were involved in agonistic interactions. Additionally, seasonal variation of FGM levels was absent in unprotected habitats (albeit significantly higher overall than in individuals from protected habitats), whereas in protected areas, FGM decreased during the wet season. These results converge with previous evidence documented for this species [26], and suggest that black howler monkeys living in unprotected habitats have higher FGM levels due to increased physiological stress associated with anthropogenic disturbance, within-group competition for resources and possibly food scarcity.

Black howler monkeys living in unprotected habitats in Campeche face numerous anthropogenic stressors. Slash-and-burn agriculture, cattle grazing, hunting, threats and attacks by domestic dogs, and fires were observed during the study in unprotected areas. These stressors have the potential to increase GC levels [11], and none of the events were recorded in protected habitats. In addition to the presence of researchers (usually three people), one of the groups living in protected areas (Calakmul Biosphere Reserve) was visited on several occasions by small groups (three to five people) of tourists, suggesting that either contact with small groups of quiet tourists does not lead to increases in FGM of black howler monkeys living in protected areas, or that we were unable to detect such effect. The fact that a positive relationship between GCs levels and intensity of tourist visitation in protected areas has been found in the majority of studies that have addressed this subject in other species [13], [40], [41] suggests that further research is required to understand the responses of black howler monkeys to the presence of tourists in protected areas [42].

Contrary to our expectation, the patterns of agonism were similar between habitat types, but in unprotected habitats individuals showed stronger FGM output to participation in agonistic interactions than individuals in protected habitats. Actually, when black howler monkeys living in unprotected habitats were not involved in agonistic interactions, their mean FGM levels, although still higher than in protected habitats, were more similar to FGM levels of individuals from protected habitats than to FGM in weeks with agonism. Therefore, in unprotected habitats black howler monkeys present higher FGM responsiveness to within-group competition. Social interactions have the potential to represent strong stressors, because they may entail both a high degree of unpredictability and an increase in metabolic demands [43], [44]. GCs play a critical role in the stimulation of gluconeogenesis and the mobilization of amino and fatty acids from body stores [45]. Thus, changes in energetic expenditure and energy balance can affect GC production, independently of psychological variables. As a consequence, in unprotected habitats, where in addition to being exposed to anthropogenic stressors, individuals may experience frequent food-deprivation [28], it is possible that acute physical exercise, such as that associated with agonistic interactions (e.g., chases, prolonged threats), increases GC secretion [46], [47]. Still, this result should be interpreted cautiously, because agonistic interactions were overall infrequent (*ca.* 0.04 interactions/h in both habitat types; see also [26]) limiting the possibility to match temporarily FGM concentrations with behavioral data [48]. Future studies should account for this limitation, and include additional measures of energy expenditure (e.g., C-peptide [49]) to understand the interplay of within-group competition, energy expenditure and FGM secretion.

As observed in many vertebrates [29], [50], the FGM of black howler monkeys varied seasonally, but this variation was only detected in protected habitats, where a decrease in hormone levels occurred in the wet season. Although we did not measure food availability, it has been reported that there is a reduction in the number of fruiting trees (a preferred food resource for this primate species) during the dry season in the forests of the Yucatan Peninsula [51], suggesting that black howler monkeys may face reduced food availability during this period. Also, fruit availability is typically reduced in forest fragments compared to continuous forests [52], [53]. In a closely related howler monkey species (*A. palliata*) [54] as well as in other primate and non-primate species (e.g., elephants: [46]; Sykes' monkeys, *Cercopithecus mitis albogularis*: [55]), when food availability decreases individuals increase energy expenditure, leading to higher FGM levels. A similar effect could explain the results found in protected habitats. FGM concentrations remained high throughout the year for black howler monkeys living in unprotected habitats. Therefore, whereas in extensive protected habitats individuals may experience seasonal increases in metabolic stress, black howler monkeys living in unprotected habitats may suffer from long-term stress due to comparatively constant food scarcity and exposure to anthropogenic stressors. Because sex was not a significant predictor of variation in GC concentrations between protected and unprotected areas and a similar number of females were lactating and pregnant in both habitats, it is unlikely that these results were affected by seasonal variation in female reproductive state [54], [56].

In Campeche, all protected areas are large (mean  = 3,255.4 km^2^), whereas few large forest remnants exist elsewhere [57]. As a consequence, in this study all protected habitats corresponded to extensive forests and all unprotected habitats were small forest fragments; and in our results the effects of habitat type on behavior and FGM cannot be separated from those of habitat size. In red howler monkeys (*Alouatta seniculus*) FGM levels vary significantly between fragments, but neither size nor human impact (logging, hunting) predict such variation, suggesting that other factors besides habitat size and human activities may be more important to understand the physiological stress responses of howler monkeys living in disturbed habitats [58]. For black howler monkeys, it remains for future research to determine if, independently from habitat size, habitat protection, accompanied by a decrease in anthropogenic pressures, is sufficient to prevent increased FGM levels. A recent study conducted in the Lacandona rainforest, however, suggests otherwise. Population composition and structure of black howler monkey groups are more strongly affected by local-scale habitat metrics, such as habitat size, than by landscape-scale variables [59]. Furthermore, forest fragment size has been proposed to be the main factor constraining populations of howler monkeys living in fragmented habitats, probably because fragment size is positively related to food availability, and negatively related to anthropogenic pressures, physiological stress and parasite loads [28]. Therefore, as for other large canopy-dwelling Neotropical mammals, including primates [60], [61], the conservation of black howler monkey populations will probably depend on the protection and maintenance of large forest tracts.

In conclusion, black howler monkeys living in unprotected areas have high FGM compared to individuals living in protected areas. As persistent high GC levels may be detrimental to health and fitness [9], black howler monkey populations living in disturbed unprotected habitats may not be viable in the long-term because there is an extinction debt to be paid in these habitats [62], [63]. To test this prediction, future research should concentrate on quantifying the effects of anthropogenic disturbances on population structure and dynamics to determine population viability. Because such data will be difficult to get, as it requires long-term studies, it may be too late for the conservation of this species by the time the data would be available. Therefore, the present study supports the idea that conservation measures may need to be taken earlier.
